# The effect of the ‘Every Mind Matters’ campaign on mental health literacy: the moderating roles of socioeconomic status and ethnicity

**DOI:** 10.1093/eurpub/ckaf020

**Published:** 2025-02-25

**Authors:** Amy Ronaldson, Kia-Chong Chua, Jane Hahn, Claire Henderson

**Affiliations:** Health Service and Population Research Department, King’s College London, Institute of Psychiatry, Psychology, and Neuroscience, London, United Kingdom; Health Service and Population Research Department, King’s College London, Institute of Psychiatry, Psychology, and Neuroscience, London, United Kingdom; Division of Psychiatry, University College London, London, United Kingdom; Health Service and Population Research Department, King’s College London, Institute of Psychiatry, Psychology, and Neuroscience, London, United Kingdom

## Abstract

We previously reported short-lived improvements in mental health literacy following the Every Mind Matters campaign, followed by a return to baseline levels. In this study, we aimed to examine whether either socioeconomic status or ethnicity moderated these improvements. We conducted regression analyses on a nationally representative, repeated cross-sectional dataset of nine survey waves from September 2019 to March 2022. Interaction terms (ethnicity*wave, socioeconomic status*wave) were entered into regression models to assess the moderating effect of these variables. Where significant interactions emerged, we obtained marginal estimates and plotted them for ease of interpretation. We found no evidence that improvements seen in mental health literacy following the launch of Every Mind Matters were moderated by ethnicity or socioeconomic status. Over time, there was some evidence of lower scores relating to symptoms recognition, knowledge of actions to improve mental health, and desire for social distance (stigma) among adults of lower socioeconomic status, which converged again for symptom recognition. These findings suggest that while a web resource can empower people and improve mental health literacy, in relation to ethnicity and socioeconomic status, it may be that while this can avoid a widening of inequalities it is insufficient to lead to a narrowing of them.

## Introduction

As internet access has increased, web-based interventions to promote on self-care and/or help seeking for mental health problems have proliferated, making use of the evidence base for increasing these actions and themselves showing promise [1, 2]. To promote these types of action in relation to mental health on the part of adults, Public Health England (PHE) developed a suite of mental health digital support resources and a promotional campaign, Every Mind Matters, which was launched in October 2019. The digital resources comprise National Health Service (NHS)-assured content covering guidance on actions that people and the public can take to address the four most common sub-clinical mental health concerns: stress, anxiety, low mood, and sleep problems. Amongst these digital resources was the ‘Mind Plan’, which is an online questionnaire based on the Warwick–Edinburgh Mental Wellbeing Scale [3], and it assesses the wellbeing of individuals and provides them with a tailored set of self-care actions to help them care for their mental health. By providing and encouraging the use of these digital resources PHE’s aim was to help prevent common mental health conditions from worsening and requiring NHS intervention.

Based on a pilot in the two Midlands regions of England in October 2018, PHE moved from a strategy that focussed on providing information about common mental health disorders to one that delivers and promotes evidence-based digital resources to facilitate self-care action for sub-clinical mental health problems for the national launch in October 2019. A second campaign under the Every Mind Matters banner across January/February 2020 encouraged people to talk openly about their mental health. A third campaign ran during April/May 2020 with a specific focus on promoting actions for people to take to care of their mental health during the first COVID-19 lockdown in response to a ministerial request. The fourth campaign in September/October 2020 changed direction to target parents rather than adults, with the aim of encouraging and supporting them to take action to look after the mental health of their children. This campaign strategy was complemented by advertising targeted directly at teenagers to help them look after their own mental health. Web analytics showed that between 7th October 2019 and 28th February 2021 the Mind Plan was completed 3 110 763 times.

We previously reported the results of our evaluation of the effectiveness of Every Mind Matters for the general adult population over the course of this time period [4]. Our approach to evaluation was based on the broad definition of mental health literacy by Kutcher [5] using the following outcomes: (1) symptom recognition and knowledge for symptom management of stress, anxiety, and depression, (2) mental health vigilance, (3) sleep-literacy, (4) self-efficacy, and (5) stigma related to mental disorders. Mental health vigilance, i.e. watching out for difficulties, fits into Kutcher’s definition of mental health literacy as it is needed for the use of relevant knowledge in the maintenance of every day mental health and wellbeing [6]. It is distinguished from hypervigilance, which involves nervous system dysregulation. Our results indicated a short-lived improvement in knowledge for managing one’s own mental health problems, sleep-literacy, and ability to promote one’s own mental health in the general population. We saw relatively better outcomes specifically in relation to knowledge of managing one’s own mental health, ability to seek help, and stigma related to mental health problems for those who were aware of the campaign. We saw lower levels of these outcomes as the covid-19 pandemic progressed, suggesting negative impacts of the pandemic, the ensuing restrictions, and the economic consequences.

Consistent with all PHE campaigns, the campaigns promoting Every Mind Matters were designed to reach people in lower socioeconomic groups, with the aim of avoiding the exacerbation of existing mental health inequalities [7], and ideally reducing them. The extent to which the effects of a public mental health intervention such as this is moderated by socioeconomic status is therefore an important question for its evaluation. It is also important to assess the relative effectiveness for minoritized groups of campaigns aimed at the general population. As it became evident that poorer minoritized ethnic groups were disproportionately affected by the negative impacts of the covid-19 pandemic [8], we aimed in this study to examine whether either socioeconomic status or ethnicity moderated the previously reported impact of Every Mind Matters on mental health literacy [4].

## Methods

### Data source and study design

Members of the general public in England responded to an online survey prior to the launch of Every Mind Matters in 2019. They were recruited from a market research panel maintained by YouGov (https://yougov.co.uk). This survey was repeated at eight further timepoints after the national launch. YouGov used quota sampling to create a sample of 20 000 participants demographically representative of adults living in households in England. New samples were drawn at each survey wave.

In this repeated measures, cross-sectional study, we used data from the nine survey waves: (1) September 2019, *n* = 3000; (2) October 2019, *n* = 2000; (3) November 2019, *n* = 2000; (4) January 2020, *n* = 2000; (5) March 2020, *n* = 3000; (6) September 2020, *n* = 2000; (7) March 2021, *n* = 2000; (8) September 2021, *n* = 2000; and (9) March 2022, *n* = 2000. We used population survey weights provided by YouGov.

### Exposures: ethnicity and socioeconomic status

Data on ethnicity and socioeconomic status were collected as part of each survey. Ethnicity was grouped into White, Mixed-White, Asian, Black, and Other based on UK census categories. Small sample sizes precluded us from including more granular ethnicity categories. Socioeconomic status was categorized using the Market Research Society’s classification system into groups based on the occupation of the chief earner in each household surveyed: AB = professional/managerial; C1 = other on-manual; C2 = skilled manual; and DE = semi-/unskilled manual.

### Outcomes

Recognition of symptoms (stress, anxiety, depression) was measured using the Mental Health Literacy—Knowledge for Recognition scale (MHL-REC). Management of stress, anxiety, and depression was measured using the Mental Health Literacy—Knowledge for Management scale (MHL—ACT) [6]. The MHL-REC comprises three identical sets of nine items and asks participants to identify whether these experiences were a symptom of stress, anxiety, or depression. Participants choose a response for each condition or the option ‘none of these’. Scores range from 0 to 9 for each condition, with higher scores indicating higher levels of knowledge for recognition. The MHL-ACT asks whether each of seven actions could help with reducing stress, anxiety, and depression. The response format is the same as the MHL-ACT and scores can range from 0 to 7 for each condition, with higher scores indicating higher levels of knowledge for management. Both scales have been evaluated for construct validity [6].

Mental health vigilance was measured using the Mental Health Vigilance scale (MHL-VIG) [6]. The MHL-VIG consists of 12 items that assess personal views about mental health. These personal attitudes foster vigilance and use of relevant knowledge for maintaining mental health in daily life. The response options are ‘strongly disagree’, ‘disagree’, ‘neutral’, ‘agree’, and ‘strong agree’. Higher scores indicate a healthier attitude towards maintaining one’s mental health. MHL-VIG demonstrated good score reliability for discriminating a large range of individual differences. MHL-VIG data also demonstrated structural validity and convergent validity with constructs related to MHL such as symptom recognition, symptom management, and mental health related stigma.

Sleep-related beliefs were measures using the Sleep Beliefs Scale (SBS) [9]. This scale comprises 20 items which measure three factors: ‘sleep-incompatible behaviours’, ‘sleep–wake cycle behaviours’, and ‘thoughts and attitudes to sleep’. The total score can range between 0 and 20 and the scale shows good internal consistency (Cronbach’s alpha = 0.71) [9].

A subscale from the Mental Health Literacy Scale (MHLS) was used to measure help-seeking self-efficacy [10] and a subscale from the Self-Rated Abilities for Health Practices Scale (SRAHPS) was used to measure psychological wellbeing self-efficacy [11]. The help-seeking subscale of the MHLS asks participants to rate four statements of confidence in seeking help (e.g. ‘I am confident that I know where to seek information about mental illness’). Items on the scale are responded to using a Likert scale with five options (‘strongly disagree’, ‘disagree’, ‘neither agree nor disagree’, ‘agree’, ‘strongly agree’). The MHLS has good internal consistency (Cronbach’s alpha = 0.87) and good test–retest reliability [10]. The psychological wellbeing subscale from the SRAHPS comprises seven items which ask how well the respondent is able to do things that promote their mental health (e.g. ‘change things in my life to reduce stress’). Items on the scale are responded to using a Likert scale with five options (‘not at all’, ‘a little’, ‘somewhat’, ‘mostly’, and ‘completely’) and the subscale demonstrates high internal consistency (Cronbach’s alpha = 0.90) and moderate test–retest reliability (*r* = 0.63) [11].

Stigma towards people with mental disorders was measured using the intended behaviour items from the Reported and Intended Behaviour Scale (RIBS) [12]. These items assess desire for social distance by asking whether a respondent would be willing to interact with someone with mental health problems in the future in four different contexts (living with, working, living nearby, continuing a relationship). Items on the scale are responded to using a five-point Likert scale (‘agree strongly’, ‘agree slightly’, ‘neither agree nor disagree’, ‘disagree slightly’, ‘disagree strongly’). Higher RIBS (ranging from 4 to 20) scores indicate more desire for social distance, i.e. more stigma. RIBS showed strong consensus validity as rated by service users, consumers, and international experts in stigma. It also demonstrated good internal consistency (Cronbach’s alpha = 0.85) and moderate to substantial test–retest reliability (*r* = 0.75).

### Covariates

Age and gender were collected as part of each survey and were included as covariates in all statistical models. Government office region is the lowest level information on participants’ location as described by the UK Government’s Office for National Statistics (ONS) and was also included as a covariate (nine regions: North East, North West, Yorkshire and Humber, East Midlands, West Midlands, East, South East, South West, London).

Survey respondents were also asked about campaign awareness. Television, radio, and web advertisements were shown for the October 2019, November/December 2019, January 2020, March 2020, September 2020, and March 2021 campaign bursts. Awareness was assessed using the question ‘Do you remember seeing this ad recently?’. Those who indicated that they had seen the content or something similar were categorized as being aware of the EMM campaign. Campaign awareness was not measured at waves in September 2019, September 2021, or March 2022 as these surveys did not coincide with an advertisement. EMM web resource use was measured using the item ‘Before taking this survey, have you visited the Every Mind Matters website?’. Respondents answering ‘Yes’ were categorized as having visited the website. Campaign awareness and EMM web resource use were not included as covariates in statistical models, but were used to describe the sample.

### Statistical analysis

To explore whether ethnicity and socioeconomic status moderated differences in mental health literacy over time, we used multivariable linear regression models with dummy variables representing seven survey waves of data collection to test the effect of time on all outcomes. Interaction terms (ethnicity*wave, socioeconomic status*wave) were entered into models to assess the moderating effect of these variables. For the purposes of interpretation, ethnicity and socioeconomic status were collapsed into binary variables before creating interaction terms. Specifically, ethnicity was recategorized as white and minoritized, and socioeconomic status was recategorized as AB/C1 and C2/DE, consistent with the targeting used by PHE for the campaigns. All models were adjusted for age, gender, and government office region. Where ethnicity*wave was entered into models, we adjusted for socioeconomic status. Where socioeconomic status*wave was entered into models, we adjusted for ethnicity. Where significant interactions emerged, we obtained marginal estimates and plotted them for ease of interpretation. All analyses were performed using STATA 18.

### Ethics

The King’s College London Psychiatry, Nursing and Midwifery Research Ethics Subcommittee exempted analysis of these survey data as secondary analysis of anonymized data.

## Results

### Sample characteristics

There was a total of 21 131 participants over nine waves of data collection. After 696 participants were excluded due to not providing their ethnicity, the final analytical sample comprised 20 435 participants. Sample characteristics are provided in Table 1. The largest proportion of respondents were consistently 55 or older, female, white, and from the AB socioeconomic group. The majority of respondents were from London and the South East.

**Table 1.** Sample characteristics

	September 2019 *N* = 3167	September/November 2019 *N* = 2097	November/December 2019 *N* = 2024	January 2020 *N* = 2010	March 2020 *N* = 2952	September 2020 *N* = 1995	March 2021 *N* = 2100	September 2021 *N* = 2121	March 2022 *N* = 1969	Total *N* = 20 435
	*N* (%)	*N* (%)	*N* (%)	*N* (%)	*N* (%)	*N* (%)	*N* (%)	*N* (%)	*N* (%)	*N* (%)
Age										
18–24	222 (7.0)	144 (6.9)	156 (7.7)	137 (6.8)	265 (9)	200 (10)	234 (11.1)	218 (10.3)	187 (9.5)	1763 (8.6)
25–34	593 (18.7)	347 (16.5)	349 (17.2)	371 (18.5)	533 (18.1)	332 (16.6)	357 (17)	361 (17)	316 (16)	3559 (17.4)
35–44	564 (17.8)	395 (18.8)	338 (16.7)	365 (18.2)	500 (16.9)	356 (17.8)	338 (16.1)	371 (17.5)	319 (16.2)	3546 (17.4)
45–54	577 (18.2)	437 (20.8)	357 (17.6)	366 (18.2)	541 (18.3)	367 (18.4)	404 (19.2)	385 (18.2)	371 (18.8)	3805 (18.6)
55+	1211 (38.2)	774 (36.9)	824 (40.7)	771 (38.4)	1113 (37.7)	740 (37.1)	767 (36.5)	786 (37.1)	776 (39.4)	7762 (38)
Gender										
Male	1467 (46.3)	956 (45.6)	979 (48.4)	937 (46.6)	1342 (45.5)	914 (45.8)	985 (46.9)	1025 (48.3)	921 (46.8)	9526 (46.6)
Female	1700 (53.7)	1141 (54.4)	1045 (51.6)	1073 (53.4)	1610 (54.5)	1081 (54.2)	1115 (53.1)	1096 (51.7)	1048 (53.2)	10909 (53.4)
Ethnicity										
White	2736 (86.4)	1844 (87.9)	1758 (86.9)	1751 (87.1)	2596 (87.9)	1722 (86.3)	1786 (85)	1800 (84.9)	1690 (85.8)	17683 (86.5)
Mixed white	85 (2.7)	42 (2)	56 (2.8)	64 (3.2)	87 (2.9)	51 (2.6)	60 (2.9)	58 (2.7)	37 (1.9)	540 (2.6)
Asian	193 (6.1)	129 (6.2)	122 (6)	107 (5.3)	153 (5.2)	115 (5.8)	149 (7.1)	165 (7.8)	139 (7.1)	1272 (6.2)
Black	66 (2.1)	38 (1.8)	45 (2.2)	50 (2.5)	63 (2.1)	57 (2.9)	53 (2.5)	41 (1.9)	49 (2.5)	462 (2.3)
Other	87 (27)	44 (2.1)	43 (2.1)	38 (1.9)	53 (1.8)	50 (2.5)	52 (2.5)	57 (2.7)	54 (2.7)	478 (2.3)
Socioeconomic status										
AB	1497 (47.3)	1014 (48.4)	806 (39.8)	931 (46.3)	1301 (44.1)	929 (46.6)	1111 (52.9)	823 (38.8)	833 (42.3)	9245 (45.2)
C1	760 (24.0)	509 (24.3)	549 (27.1)	486 (24.2)	752 (25.5)	514 (25.8)	426 (20.3)	605 (28.5)	380 (19.3)	4981 (24.4)
C2	373 (11.8)	235 (11.2)	294 (14.5)	244 (12.1)	395 (13.4)	249 (12.5)	246 (11.7)	281 (13.2)	316 (16)	2633 (12.9)
DE	537 (17.0)	339 (16.2)	375 (18.5)	349 (17.4)	504 (17.1)	303 (15.2)	317 (15.1)	412 (19.4)	440 (22.3)	3576 (17.5)
Government office region										
North East	152 (4.8)	106 (5.1)	82 (4.0)	91 (4.5)	139 (4.7)	89 (4.5)	98 (4.7)	98 (4.6)	93 (4.7)	948 (4.6)
North West	402 (12.7)	283 (13.5)	254 (12.5)	230 (11.4)	363 (12.3)	250 (12.5)	263 (12.5)	275 (13.0)	249 (12.6)	2569 (12.6)
York and Hum	327 (10.3)	198 (9.4)	198 (9.8)	197 (9.8)	295 (10.0)	190 (9.5)	192 (9.1)	206 (9.7)	186 (9.4)	1989 (9.7)
East Midlands	277 (8.7)	167 (8.0)	176 (8.7)	162 (8.1)	234 (7.9)	170 (8.5)	164 (7.8)	178 (8.4)	166 (8.4)	1694 (8.3)
West Midlands	323 (8.7)	189 (9.0)	219 (10.8)	182 (9.0)	293 (9.9)	201 (10.1)	175 (8.3)	229 (10.8)	206 (10.5)	2017 (9.9)
East	351 (11.1)	246 (11.7)	226 (11.2)	220 (10.9)	333 (11.3)	212 (10.6)	22 (10.6)	242 (11.4)	226 (11.5)	2278 (11.1)
South East	516 (16.3)	349 (16.6)	338 (16.7)	342 (17.0)	503 (17.0)	329 (16.5)	367 (17.5)	356 (16.8)	338 (17.2)	3438 (16.8)
South West	329 (10.4)	236 (11.2)	210 (10.4)	225 (11.2)	319 (10.8)	205 (10.3)	227 (10.8)	218 (10.3)	195 (9.9)	2164 (10.6)
London	490 (15.5)	323 (15.4)	321 (15.9)	361 (18.0)	473 (16.0)	349 (17.5)	392 (18.7)	319 (15.0)	310 (15.7)	3338 (16.3)
Campaign awareness										
Aware	N/A	906 (43.2)	808 (39.9)	720 (35.8)	1038 (35.2)	361 (18.1)	604 (27.9)	N/A	N/A	4423 (33.6)
Unaware	N/A	1191 (56.8)	1216 (60.1)	1290 (64.2)	1914 (64.8)	1634 (81.9)	1564 (72.1)	N/A	N/A	8755 (66.4)
Web visit										
Yes	N/A	88 (4.2)	86 (4.2)	63 (3.1)	48 (1.6)	53 (2.7)	99 (4.7)	N/A	N/A	437 (3.3)
No	N/A	2009 (95.8)	1938 (95.8)	1947 (96.9)	2904 (98.4)	1942 (97.3)	2001 (95.3)	N/A	N/A	12741 (96.7)

### The moderating effect of ethnicity and socioeconomic status

Interactions between study wave and ethnicity were not associated with any outcome indicating that change over time in all outcomes did not differ according to ethnicity (see Supplementary Table S1).

**Figure 1.**
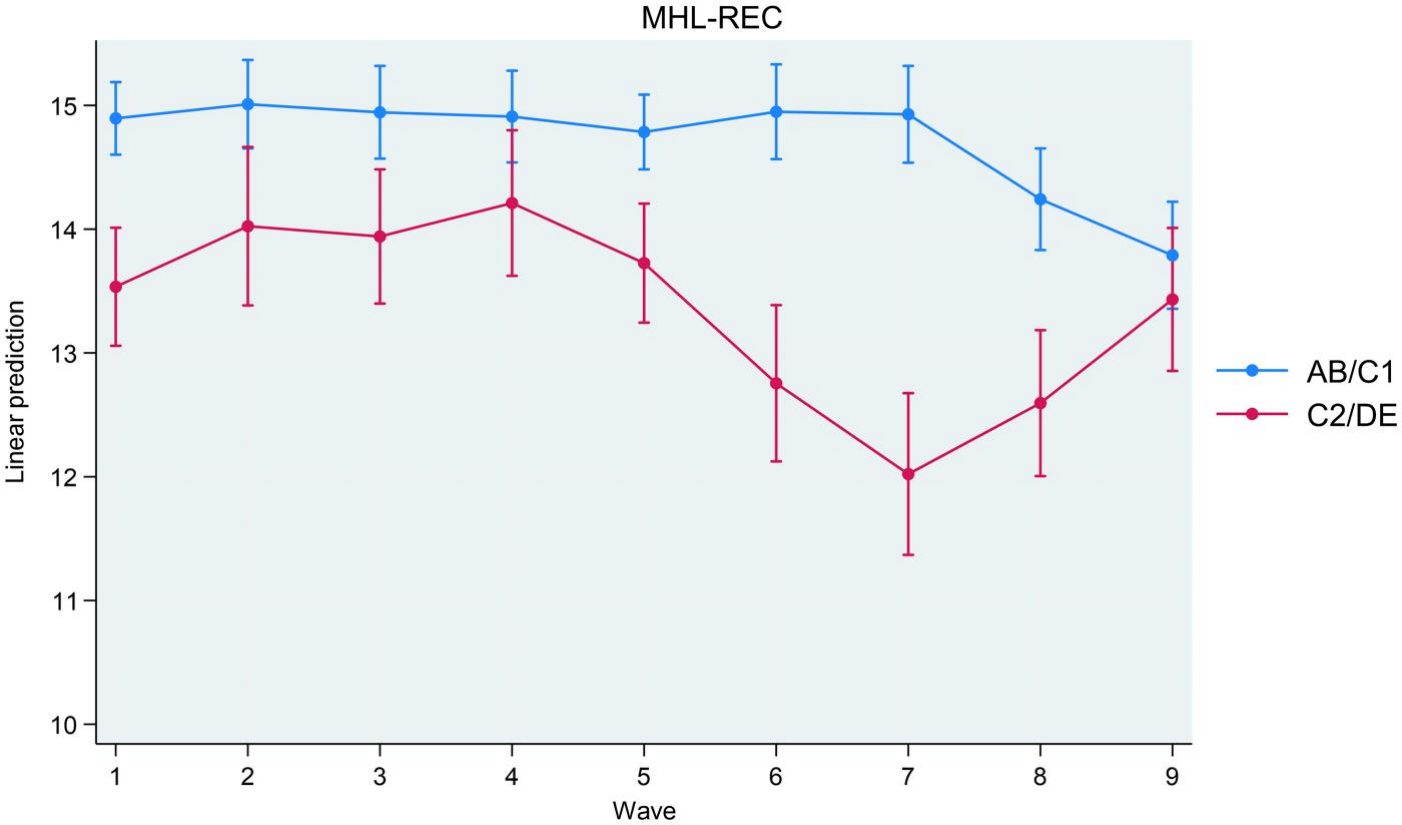
Marginal estimates and associated confidence intervals for SES*wave interaction on knowledge for recognition (MHL-REC) scores. Significant differences between people from the AB/C1 group and the C2/DE group are indicated by non-overlapping confidence intervals.

**Figure 2.**
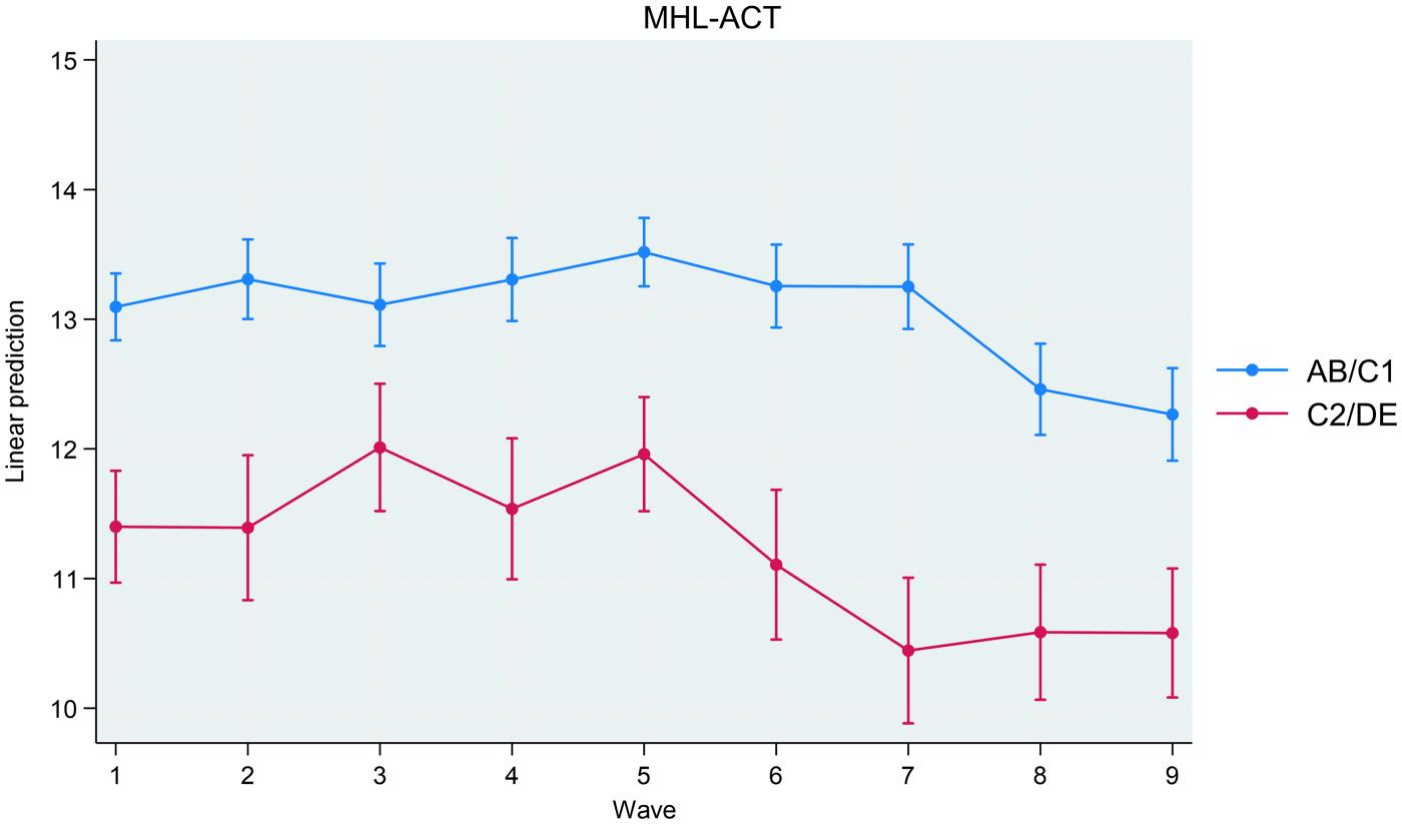
Marginal estimates and associated confidence intervals for SES*wave interaction on knowledge for management (MHL-ACT) scores. Significant differences between people from the AB/C1 group and the C2/DE group are indicated by non-overlapping confidence intervals.

**Figure 3.**
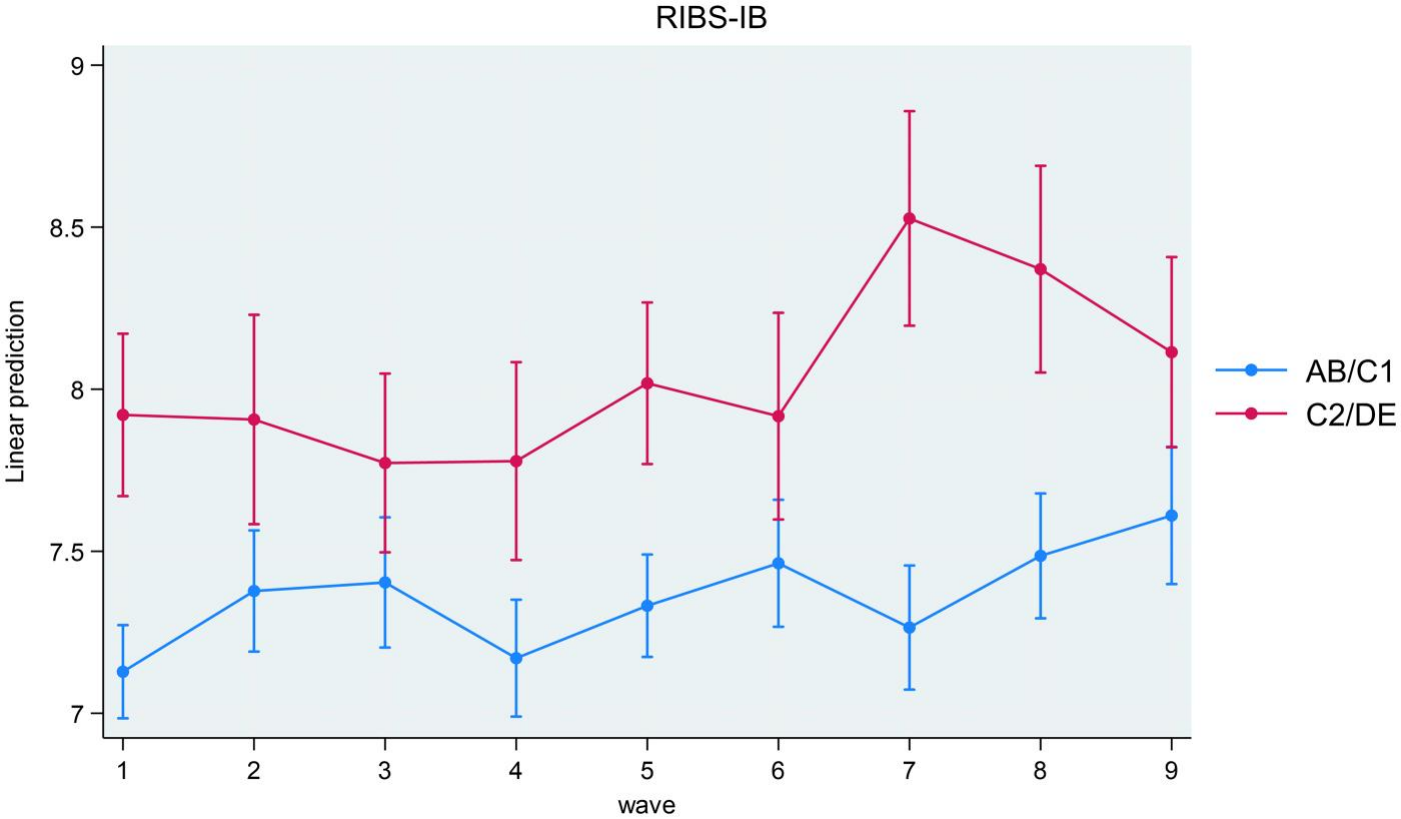
Marginal estimates and associated confidence intervals for SES*wave interaction on intended behaviour items of the Reported and Intended Behaviour Scale (RIBS). Significant differences between people from the AB/C1 group and the C2/DE group are indicated by non-overlapping confidence intervals.

Interactions between study wave and socioeconomic status were associated with MHL-REC (*P* < .001), MHL-ACT (*P* = .029), and RIBS-IB (*P* = .031). Interactions between study wave and socioeconomic status were not associated with any other outcome. Coefficients for interactions are present in Supplementary Table S2. Marginal estimates for MHL-REC (recognition of symptoms), MHL-ACT (knowledge for management), and RIBS-IB (desire for social distance) are plotted in Figs 1–3 for interpretation. MHL-REC scores were higher for people from the AB/C1 compared to the C2/DE socioeconomic group over time, with the disparity between these two groups becoming most pronounced at Wave 7 (March 2021). MHL-REC scores for each group converged at Wave 4 (January 2020) and Wave 9 (March 2022). MHL-ACT scores were consistently higher for people from the AB/C1 compared to the C2/DE socioeconomic group over time with this disparity becoming widest at Wave 6 (September 2020) and Wave 7 (March 2021). RIBS-IB scores were higher in the C2/DE socioeconomic group compared to the AB/C1 group over time, with some convergence seen at Wave 3 (November 2019) and Wave 6 (September 2020).

## Discussion

Regarding the short-term improvements in the outcomes following the launch of Every Mind Matters that we previously reported, we found no evidence for differential effectiveness by ethnicity or socioeconomic status. Over time, we found some evidence of relatively lower scores in relation to symptom recognition, knowledge of actions to improve mental health, and stigma as measured by desire for social distance among adults of lower socioeconomic status, which only converged again for symptom recognition. In keeping with the interpretation of the lower scores in later waves as reflecting the impact of the covid-19 pandemic on the normalization of worse mental health and difficulties in taking self-care action or help-seeking, these divergences may reflect a greater impact of the pandemic on lower socioeconomic groups in terms of these difficulties. Increased desire for social distance may reflect fears or assumptions about people perceived as likely to pose a risk of contagion; given higher rates of infection in areas of greater deprivation this desire may have been stronger over this period.

### Strengths and limitations

Our survey used a range of outcomes based on an accepted definition of mental health literacy, measured with scales with established psychometric properties that were suitable to assess the effects of the Every Mind Matters resource within the general adult population. However, the sampling for the surveys was not designed to compare changes over time among different demographic groups; this would have entailed oversampling of minoritized groups. More generally there are design limitations which mean we cannot establish temporality between the campaign and the outcomes.

We do not know whether respondents benefitted from the campaign or whether people who were more mental health literate are more likely to remember the campaign. Regarding the digital resources, the population survey design does not measure the impact of their use as opposed to the population level impact of the campaign, as the subsample of people who visited the campaign website or interacted with the digital resources are too small a proportion within the survey sample. Evaluation of the digital resource itself would require pre–post measurement of outcomes for resource users and a control group.

Another limitation is that the study design is a repeated cross-sectional study. Our analyses used respondents from the baseline survey wave prior to the launch of the campaign as the comparator group. Our analyses operate under the assumption that respondents from the baseline survey wave are comparable in demographic and unobserved characteristics to the respondents from subsequent survey waves. While we use quota sampling to ensure that respondents from all survey waves are nationally representative of demographics of private households in England, there may be unobserved characteristics that differ between waves other than exposure to the Every Mind Matters campaign over time. Further, the variation that we see over time may be due to standard regressions towards the mean phenomenon.

Finally, while quota sampling ensures that subgroups in the population are represented within the study sample, it does not randomly draw samples from the population [13]. Therefore, the results may not be entirely generalizable to the adult population in England.

### Implications

The high level of engagement with the Every Mind Matters web resources reflects the success of the campaign in addressing one of the most commonly expressed reasons for not seeking help for poor mental health, namely the desire to address this oneself [14]. The early results of this study also suggest that the campaign succeeded in avoiding any exacerbation of pre-existing inequalities in mental health literacy by socioeconomic status or race/ethnic group. The subsequent divergences in some aspects of mental health literacy, with relatively lower scores for people with lower socioeconomic status, may suggest relatively greater negative impacts of the pandemic on these groups. However, the pre-existing and continued demographic disparities in recognition of symptoms and knowledge of actions that can be taken may reflect ongoing disparities in the extent to which people normalize poor mental health and feel unable to act on it.

## Conclusions

Our qualitative evaluation identified that some users of the web resource felt frustration that it did not sufficiently acknowledge the impact on mental health of social and environmental problems [15] which they could not address, such as poor housing and global heating. Taken together, these findings suggest that while a web resource can empower people who prefer to focus on self-care rather than seek help, or have not benefitted from professional help, it may be that while this can avoid a widening of inequalities it is insufficient to lead to a narrowing of them in relation to race/ethnicity and socioeconomic status. This suggests the need to consider the evidence base for, and implementation of, those interventions and policies directed at social and environmental problems and targeted to people in lower socioeconomic circumstances and minoritized ethnic groups.

### Lived experience commentary

The Every Mind Matters (EMM) campaign, aimed at improving mental health literacy through digital resources, has shown some success. However, this study shows there was no differential impact of the campaign by ethnicity or socioeconomic status. While this at least indicates that the campaign maintained a broad appeal without increasing inequalities, it then raises questions about its impact on the communities who might be disproportionately in need of support. Addressing minoritized groups' specific needs and barriers remains essential.

Mental health issues are compounded by systemic issues such as unemployment, poor housing, discrimination, and lack of access to culturally competent care. EMM’s digital approach, while beneficial, may need to be supplemented with targeted support and resources to address these deeper issues for specific communities.

For example, combining digital information with face-to-face interactions and peer support could enhance the campaign's effectiveness. Many people value the trust and safety of personal interactions, which online platforms alone may not provide. Creating safe spaces where individuals can share their experiences and receive peer support could complement digital resources, making mental health literacy efforts more impactful and encouraging communities to be ambitious in their expectations of good mental health.

The campaign's efforts to target lower socioeconomic groups are commendable, though the engagement data suggests a need for improved approaches. By involving diverse groups from the outset in the design and evaluation of public health campaigns, their reach and relevance can be significantly improved. While EMM has made notable strides in raising mental health awareness, there are opportunities to enhance its impact on Black and minoritized communities. Future campaigns should incorporate inclusive strategies that address both cultural contexts and systemic barriers, ensuring that mental health resources are accessible, relatable, and effective for everyone.

## Supplementary Material

ckaf020_Supplementary_Data

## Data Availability

The data underlying this article will be shared on reasonable request to the corresponding author.
