# Supplementary material for: The effect of the ‘Every Mind Matters’ campaign on mental health literacy: the moderating roles of socioeconomic status and ethnicity
Source: Eur J Public Health. 2025 Feb 25;35(2):366–72. doi: 10.1093/eurpub/ckaf020 (PMC11967896; doi:10.1093/eurpub/ckaf020)
Supplement: ckaf020_Supplementary_Data [file ckaf020_supplementary_data.docx]

**The effect of the ‘Every Mind Matters’ campaign on mental health literacy: The moderating roles of socioeconomic position and ethnicity**

**Supplementary Material**

| **Table S1.** Associations between wave*ethnicity and all outcomes | | | | | | | |
| --- | --- | --- | --- | --- | --- | --- | --- |
|  | **MHL-REC** | **MHL-ACT** | **MHL-VIG** | **SBS** | **MHLS subscale** | **SRAHPS subscale** | **RIBS – IB** |
|  | *p value* | *p value* | *p value* | *p value* | *p value* | *p value* | *p value* |
| **Overall associations** | 0.745 | 0.222 | 0.724 | 0.777 | 0.360 | 0.180 | 0.804 |
| **Wave*ethnicity** | *Β (95% CI)* | *Β (95% CI)* | *Β (95% CI)* | *Β (95% CI)* | *Β (95% CI)* | *Β (95% CI)* | *Β (95% CI)* |
| **2** | -0.63 (-1.91 to 0.65) | -0.36 (-1.58 to 0.86) | 0.71 (-0.62 to 2.04) | 0.08 (-0.77 to 0.93) | 0.49 (-0.17 to 1.15) | 0.39 (-0.74 to 1.53) | -0.48 (-1.13 to 0.17) |
| **3** | 0.26 (-0.93 to 1.44) | -0.65 (-1.77 to 0.48) | 0.26 (-0.99 to 1.51) | 0.39 (-0.42 to 1.20) | -0.01 (-0.64 to 0.62) | -0.45 (-1.59 to 0.69) | 0.12 (-0.53 to 0.76) |
| **4** | -0.43 (-1.68 to 0.83) | -0.25 (-1.43 to 0.93) | 0.92 (-0.37 to 2.21) | 0.20 (-0.61 to 1.01) | 0.49 (-0.14 to 1.11) | 1.10 (0.01 to 2.20)* | -0.01 (-0.71 to 0.69) |
| **5** | 0.33 (-0.76 to 1.43) | -0.49 (-1.54 to 0.56) | **-** | 0.21 (-0.51 to 0.92) | 0.22 (-0.37 to 0.81) | -0.35 (-1.33 to 0.63) | -0.19 (-0.80 to 0.42) |
| **6** | 0.34 (-0.94 to 1.63) | 0.58 (-0.59 to 1.76) | **-** | 0.53 (-0.21 to 1.28) | **-** | 0.09 (-1.00 to 1.18) | 0.18 (-0.50 to 0.87) |
| **7** | 0.00 ( -1.27 – 1.27) | 0.02 (-1.14 to 1.18) | 0.35 (-0.88 to 1.58) | 0.53 (-0.25 to 1.31) | **-** | -0.10 (-1.18 to 0.98) | 0.02 (-0.65 to 0.69) |
| **8** | -0.17 (-1.39 to 1.04) | -0.91 (-2.03 to 0.20) | -0.15 (-1.37 to 1.08) | 0.64 (-0.13 to 1.42) | **-** | 0.60 (-0.43 to 1.63) | -0.01 (-0.68 to 0.66) |
| **9** | -0.65 (-1.91 to 0.62) | -1.10 (-2.23 to 0.03) | 0.10 (-1.16 to 1.36) | 0.18 (-0.64 to 1.00) | **-** | 0.62 (-0.43 to 1.68) | 0.05 (-0.63 to 0.72) |
| *p<0.05  **p<0.001  All models adjusted for age, gender, socioeconomic position, government region  Note: ethnicity was collapsed into a binary variable (White, non-White) in order to make interaction terms with study wave more interpretable | | | | | | | |

| **Table S2.** Associations between wave*socioeconomic position (SEP) and all outcomes | | | | | | | |
| --- | --- | --- | --- | --- | --- | --- | --- |
|  | **MHL-REC** | **MHL-ACT** | **MHL-VIG** | **SBS** | **MHLS subscale** | **SRAHPS subscale** | **RIBS - IB** |
|  | *p value* | *p value* | *p value* | *p value* | *p value* | *p value* | *p value* |
| **Overall associations** | **<0.001**** | **0.029*** | 0.062 | 0.388 | 0.876 | 0.195 | **0.031*** |
| **Wave*SEP** | *Β (95% CI)* | *Β (95% CI)* | *Β (95% CI)* | *Β (95% CI)* | *Β (95% CI)* | *Β (95% CI)* | *Β (95% CI)* |
| **2** | 0.37 (-0.55 to 1.30) | -0.22 (-1.03 to 0.59) | -0.77 (-1.59 to 0.05) | -0.06 (-0.60 to 0.47) | -0.24 (-0.69 to 0.21) | -0.55 (-1.35 to 0.24) | -0.26 (-0.73 to 0.21) |
| **3** | 0.36 (-0.51 to 1.22) | 0.60 (-0.18 to 1.37) | -0.16 (-0.95 to 0.64) | -0.01 (-0.51 to 0.49) | -0.16 (-0.59 to 0.27) | -0.39 (-1.15 to 0.37) | -0.42 (-0.87 to 0.02) |
| **4** | 0.66 (-0.23 to 1.55) | -0.07 (-0.88 to 0.73) | 0.40 (-0.42 to 1.21) | 0.13 (-0.39 to 0.65) | -0.13 (-0.57 to 0.30) | -0.30 (-1.09 to 0.49) | -0.18 (-0.64 to 0.27) |
| **5** | 0.30 (-0.50 to 1.10) | 0.14 (-0.58 to 0.86) | **-** | 0.12 (-0.34 to 0.58) | -0.09 (-0.49 to 0.30) | 0.30 (-0.39 to 0.99) | -0.11 (-0.52 to 0.31) |
| **6** | -0.83 (-1.76 to 0.09) | -0.45 (-1.28 to 0.38) | **-** | -0.40 (-0.92 to 0.13) | **-** | 0.20 (-0.60 to 1.00) | -0.34 (-0.81 to 0.13) |
| **7** | **-1.55 (-2.49 to -0.60)**** | **-1.11 (-1.93 to -0.29)*** | -0.51 (-1.31 to 0.29) | -0.38 (-0.91 to 0.15) | **-** | -0.46 (-1.25 to 0.33) | 0.47 (-0.01 to 0.95) |
| **8** | -0.29 (-1.20 to 0.62) | -0.18 (-0.98 to 0.63) | 0.18 (-0.61 to -0.97) | 0.13 (-.040 to 0.66) | **-** | -0.18 (-0.93 to 0.58) | 0.09 (-0.38 to 0.56) |
| **9** | **1.00 (0.09 to 1.92)*** | 0.01 (-078 to 0.80) | 0.41 (-0.37 to 1.20) | 0.11 (-0.41 to 0.63) | **-** | -0.65 (-1.40 to 0.10) | -0.29 (-0.75 to 0.17) |
| *p<0.05  **p<0.001  All models adjusted for age, gender, ethnicity, government region  Note: SEP was collapsed into a binary variable (AB/C1, C2/D1) in order to make interaction terms with study wave more interpretable | | | | | | | |
